# The Effect of Passive Dehydration on Phase Angle and Body Composition: A Bioelectrical Impedance Analysis

**DOI:** 10.3390/nu16142202

**Published:** 2024-07-10

**Authors:** Jorge A. Aburto-Corona, Juan J. Calleja-Núñez, José Moncada-Jiménez, José Antonio de Paz

**Affiliations:** 1 Doctoral School Physical Activity and Sports Sciences, University of Leon, 24007 Leon, Spain; 2 Faculty of Sports, Autonomous University of Baja California, Tijuana 22424, Mexico; juan.calleja@uabc.edu.mx; 3 Human Movement Sciences Research Center (CIMOHU), University of Costa Rica, San José 11501, Costa Rica; jose.moncada@ucr.ac.cr; 4 Institute of Biomedicine (IBIOMED), University of Leon, 24071 Leon, Spain; japazf@unileon.es

**Keywords:** validity, reliability, dehydration, total body water, body composition, bioimpedance analysis, BIA

## Abstract

Bioelectrical impedance analysis (BIA) is a method used to estimate body composition, and it relies mainly on the body’s water content. Insufficient body water can introduce bias to body composition scores. Purpose: To determine the effect of body weight loss elicited by passive dehydration on body composition scores, including phase angle (PhA). Methods: Twenty-five euhydrated apparently healthy and physically active men’s (age = 22.6 ± 3.3 yr.; body mass = 76.7 ± 15.9 kg; height = 172.0 ± 6.3 cm) body composition variables and PhA were measured before and after sitting quietly for 5 h in a controlled environment (26.6 ± 1.7 °C, 72 ± 4.9%RH). Results: It was found that five hours of passive dehydration caused a loss in body weight (Δ = 0.76 ± 0.34 kg, *p* < 0.05) and a decrease in body fat estimation (Δ = 0.90 ± 0.87 kg, *p* < 0.001). Additionally, an increase in ECW (Δ = 0.12 ± 0.30 L, *p* < 0.021) and PhA (Δ = 0.10 ± 0.15°, *p* < 0.005) was observed. Conclusion: Body weight loss due to passive dehydration decreased BIA-derived fat mass, and increased extracellular water and PhA in physically active and apparently healthy men. Nonetheless, these changes had a negligible effect on the accuracy of the equipment, rendering them clinically insignificant.

## 1. Introduction

**Figure 1:**
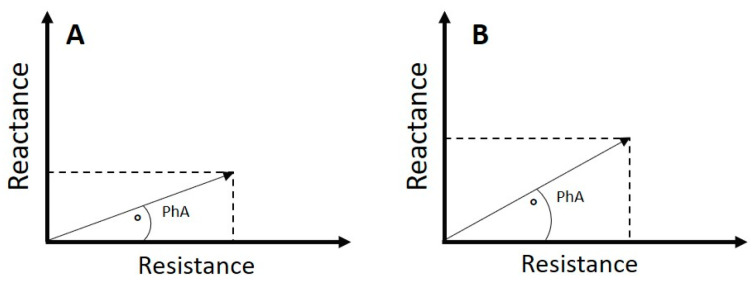
An example of phase angle (PhA) of two individuals with the same characteristics but one with a chronic disease (**A**) and the other apparently healthy (**B**).

Bio-electrical impedance analysis (BIA) is a fast, non-invasive method to estimate body composition, assessing tissue resistance and cell membrane capacitance [1,2]. Additionally, the BIA enables phase angle (PhA) calculation, a variable used to report the cell membrane’s quality and the cell’s nutritional status [3]. PhA represents the angle formed by the impedance vector (*Z*) between resistance (*R*) and reactance (*X_c_*) at a given frequency, calculated as the arctangent of the radius between resistance and reactance (PhA = [arctangent (*X_c_*/*R*)] × 180°/π) [4,5] (Figure 1A).

Recent evidence evaluated the predictive value of PhA in several diseases based on the premise that membrane integrity is critical for cell survival [6]. Thus, PhA is used as a criterion to recognize healthy cells, where a low value represents a deteriorated cell and high values indicate a cell in good condition [7] (Figure 1A,B). In fact, PhA was a good indicator for determining the severity of COVID-19 infection or predicting mortality in people with HIV [8,9].

However, determining the state of hydration before obtaining the PhA should be considered a priority since body water is the primary source of electrical current conduction in the human body [10]. Even so, many investigations evaluate variables of body composition and PhA without considering hydration state [11,12]. For instance, PhA values could vary due to changes in warm ambient temperature and relatively high humidity [13]. 

Some studies using dilution techniques indicate that changes in total body water and the distribution of intra- and extracellular fluids affect resistance and reactance, elongating the impedance vector [14,15,16]. These changes appear to limit the applicability of predictive equations generated by BIA models, directly impacting the estimation of body composition analysis, making it unreliable in clinical situations.

Thus, this study aimed to determine if acute water loss (i.e., decrease in body mass) elicited by passive dehydration under warm and humid conditions influences BIA-computed body fat, muscle mass, total body water (TBW), intracellular water (ICW), extracellular water (ECW), and PhA values in healthy physically active young adults.

## 2. Methods

### 2.1. Trial Design

We used a single-arm repeated-measures clinical trial to evaluate the effects of weight loss due to passive dehydration on phase angle and body composition through bioelectrical impedance analysis (BIA). All variables were measured at baseline (pre-test) and after five hours of exposure to a warm (26.6 ± 1.7 °C) and humid (72 ± 4.9%) environment (post-test). This study is reported following the Consolidated Standards of Reporting Trials (CONSORT) extension for non-pharmacologic treatments [17].

### 2.2. Participants 

Apparently healthy, physically active male college students were recruited through a call to participate. A total of 25 men were potentially eligible to participate (the mean environmental temperature and relative humidity of residence were 17 °C and 71%, respectively). Individuals with a medical history of metabolic disorders, poor nutritional status, hyperhidrosis, bone or muscle problems, hypo- or hypertension, or with any device or implant that may interfere with the BIA were excluded from the study. This study was performed in accordance with the ethical guidelines of the Declaration of Helsinki and were reviewed and approved by the Evaluation Committee for Research and Postgraduate Studies from the Autonomous University of Baja California, Mexico (code P-02-2023-1). All participants provided written informed consent before being allowed to participate, and information regarding the aim of this study, potential risks, and protection of the subjects’ rights were provided per the latest version of the Declaration of Helsinki [18].

### 2.3. Procedures 

Before attending the Human Motor Biosciences Laboratory, participants were asked to strictly follow the following dietary requirements: (a) to increase the fluid intake the day before to ensure proper hydration and record the consumption of 500 mL of fluid at 4:00 p.m., 7:00 p.m., and 10:00 p.m. the day before the assessment (excluding water ingested in the diet); (b) to avoid eating or drinking for at least 8 h; and (c) to avoid ingesting liquor or diuretic drinks for at least 24 h. It is important to mention that this hydration protocol was used in other studies with a similar population [19,20]. Participants were also asked not to engage in intense physical activity for at least 24 h before measurement, as previously recommended [19]. All participants attended the laboratory facilities only once (7:00 am). Upon arrival, they were asked to defecate and urinate. Urine specific gravity (USG) analysis was performed with a urinary refractometer (Atago MASTER-SUR/Nα; Tokyo, Japan). The visit to the laboratory was rescheduled if the individual’s urine showed USG values indicative of dehydration (USG > 1.020) [21]. Once it was verified that the participants were in an optimal state of hydration, the height was measured (InBody BSM 170; Seoul, Republic of Korea) following a standardized protocol [22,23].

Bioelectrical impedance analysis. Before the BIA analysis, individuals were asked to leave their wallets, purses, or coins outside the measurement site. They were asked to remove all metal objects: rings, bracelets, piercings, and hair clips. In addition, they were asked to wear clothes that did not have metal zippers, clasps, rivets, or metal accessories. Once this was carried out, the operator began measuring body composition. An octopolar bioelectrical impedance analysis (InBody 770; Seoul, Republic of Korea) was performed with the subject standing using tactile electrodes (two pairs on feet and two pairs on hands). Single-frequency (50 kHz) PhA was measured, while a multi-frequency (1, 5, 50, 250, 500, 1000 kHz) mode was used for the rest of the variables: fat mass (FM), skeletal muscle mass (SMM), TBW, ICW, and ECW. 

### 2.4. Passive Dehydration

Participants were asked to remain seated for 5 h in a room set at a temperature of 26.6 ± 1.7 °C and relative humidity of 72.0 ± 4.9%. Both the duration of sitting and the ambient temperature were selected because previous studies reported significant changes in body weight under similar conditions during passive dehydration [24,25]. During this period, participants did not ingest beverages or food, nor were they allowed to use the restroom. Participants were asked to wear shorts throughout the protocol to facilitate sweating. During this time, HR was recorded every 15 min. When the passive dehydration time was over, the participants were taken to a room with a temperature of 21.8 °C and a relative humidity of 38%. They were asked to remove excess sweat with a dry cloth and lie supine for 10 min. Subsequently, body mass (BM), FM, FFM, and PhA were re-evaluated (post-intervention). Finally, subjects were thanked for participating and provided a snack consisting of fresh fruit, a cookie, and a beverage. It is important to mention that the position in which the participants remained for the 5 h does not affect the measurement of the BIA variables [26,27]. More than 15 min in the supine position are required to affect BIA measurements.

### 2.5. Statistical Analysis 

The statistical packages IBM-SPSS, version 23 (IBM Corp., Armonk, NY, USA) and JASP version 0.18.3 (JASP Team, 2024) were used to analyze the information. Data are expressed as mean (M) and standard deviation (SD). Normality assumptions were studied, and paired sample *t*-tests or Wilcoxon tests were performed between baseline and dehydrated conditions for the variables BM, FM, SMM, TBW, ICW, ECW, and PhA. The 95% confidence interval for the mean differences (CI95%diff) was computed. For paired *t*-tests, the effect size was calculated as Cohen’s d and interpreted as small (≤0.2), moderate (0.5), and large (≥0.8) [28]. For the Wilcoxon test, the effect size is given by the matched rank biserial correlation, which is interpreted as very low (0.00 < 0.20), low (0.20 < 0.40), moderate (0.40 < 0.60), strong (0.60 < 0.80), and very strong (0.80 < 1.00) [29]. The statistical significance was set a priori at *p* < 0.05. The absolute reliability in body composition scores was studied by the typical error of the measurement (TEM), Bland–Altman plots and the coefficient of variability (CV). The TEM was computed as SDdiff/√2 (SDdiff is the standard deviation of the difference scores). The CV was computed as M/TEM × 100 and interpreted as small if ≤10% [30]. Finally, the smallest worthwhile change (SWC) in body composition scores was computed as SWC = TEM × √2 × 0.2 [31]. The Mean Absolute Percentage Error (MAPE) measures the average error between the hydrated and dehydrated conditions, indicating the accuracy of the variables (BM, FM, SMM, TBW, ICW, ECW, and PhA). 

## 3. Results

**Figure 2:**
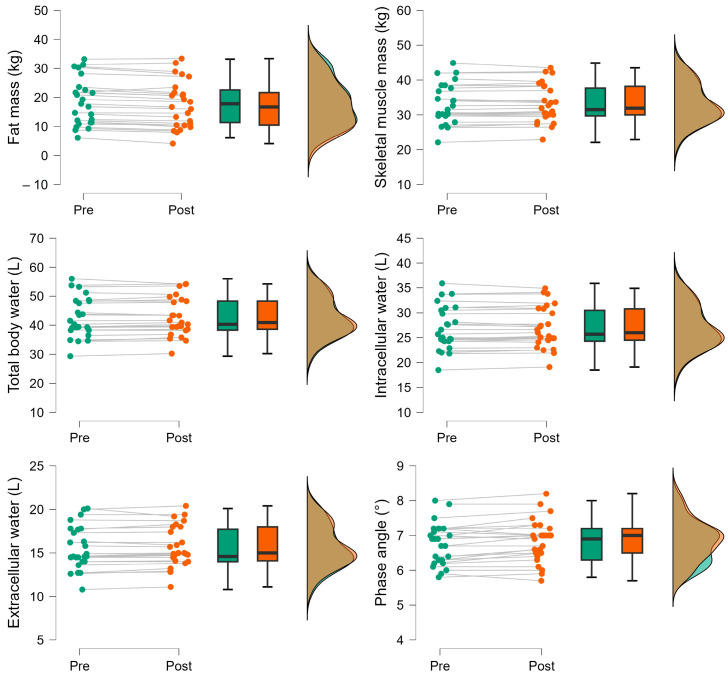
Raincloud plots showing statistically significant individual and group changes in body weight, fat mass, extracellular water, and phase angle following five hours of passive dehydration under thermal stress in males (*n* = 25).

A total of 25 male students (age = 22.6 ± 3.3 years; body mass = 76.7 ± 15.9; height 172.0 ± 6.3 cm) completed this study and were included in the final analysis. All participants arrived at the laboratory euhydrated (USG = 1.010 ± 0.006). No significant mean differences were found in SMM, TBW, and ICW (Table 1). Finding differences in weight but not in body water indicates that the BIA equipment has a measurement error. Similarly, it is possible that the equipment is not sufficiently sensitive to detect differences in these variables with such small changes in body weight. Significant mean differences were found in body mass, FM, ECW, and PhA (Table 1; Figure 2).

**Figure 3:**
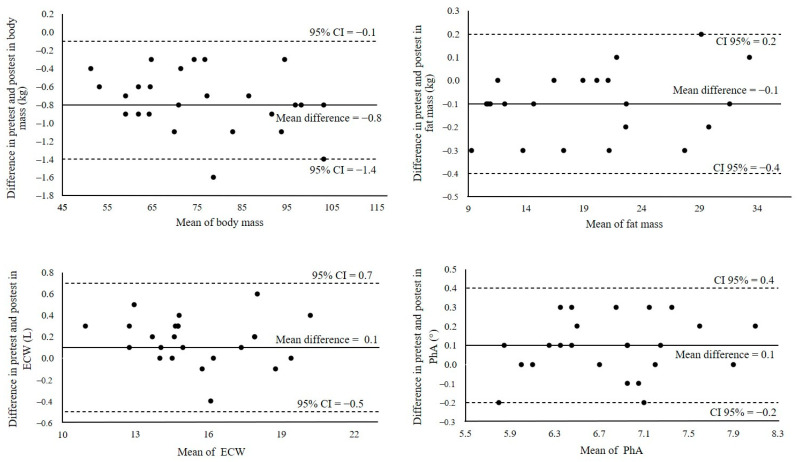
Bland–Altman plots for body mass, fat mass, extracellular water (ECW), and phase angle (PhA) between the pre-test and post-test.

Although there were differences in body weight, FM, ECW, and PhA, the Bland–Altman graph, combined with the 95% CI, indicates very good consistency in those variables (Table 1; Figure 3); in other words, the BIA equipment provides reasonably accurate information despite minimal changes in body weight caused by passive dehydration. These graphs indicate that water loss (due to passive dehydration) underestimates body weight, which is expected since the subjects remained seated in a warm environment. However, the graph shows an underestimation of body fat and an overestimation of ECW and PhA (Figure 2 and Figure 3). In other words, a loss of 0.8 kg of body weight will result in biased data in body fat (−0.1 kg), ECW (0.1 L), and PhA (0.1°) measured by BIA. These biases may arise from limitations in the formulas used within the BIA equipment, as there is no direct method to determine body fat percentage or phase angle. Nevertheless, the data distribution in the graphs was reasonably normal. 

Although there were variations in body weight, FM, ECW, and PhA between the two measurements (i.e., euhydrated and after passive dehydration), the InBody 770 BIA equipment produced reliable values. The FM had the highest CV (3.43%), while other variables showed coefficients of variation <2% (Table 2). The SWC was low, with PhA showing the lowest value (0.03° [6.73–6.79°]) and FM the highest value (0.17 kg [18.23–18.57 kg]) (Table 2). The TEM indicates that despite dehydration, the measurements are consistent in their values. In the case of ECW, the TEM is greater than the mean difference, indicating significant uncertainty in the measurement of this variable. A similar situation occurs with fat mass and PhA, where the mean differences are nearly equal to the TEM.

## 4. Discussion

This study aimed to determine if an acute reduction in body mass due to passive dehydration significantly impacts body fat, muscle mass, total body water, intracellular water, extracellular water, and phase angle measured by BIA in physically active men. The individuals remained seated for 5 h under thermal stress in a room at 26.6 °C and relative humidity of 72%. The individuals were not allowed to ingest food or liquids, defecate, or urinate during this period; therefore, the weight loss was explained mainly by body water losses (i.e., passive sweating). The most important finding was that a slight body mass loss (0.76 kg) significantly decreased fat mass by 0.9 kg and increased extracellular water by 0.1 L and phase angle by 0.1°. This finding in FM, ECW, and PhA values might have potential practical implications in some sports where athletes acutely make weight before a competition [32]. However, the coefficient of variability, the smallest worthwhile change, and the mean absolute percentage error demonstrated that the changes cannot be considered clinically relevant.

Tinsley et al. (2022) [33] found that acute water intake (11 mL/kg) increased estimated body fat (~1.3 kg), while upper body impedance increased and lower body impedance decreased. These changes in impedance introduce noise (i.e., technical error) in body composition estimation, consistent with findings reported by others attributing these changes to water redistribution among different body segments (e.g., arms, legs, trunk) or body compartments (i.e., intra- and extracellular spaces), aspects not accounted for in the algorithms used by these devices [33,34,35,36,37]. Regarding PhA, the slight increase may be associated with a decrease in FM, variables that maintain an inverse association [38].

An increase in PhA suggests improved cellular membrane integrity, which translates to better cellular health. This possible mechanism could be explained by a response to dehydration, specifically fluid exchange from the intracellular to extracellular compartment [39]. However, researchers mention that a clinically significant difference between pre-test and post-test is 0.9° for women and 1.0° for men [40]. It is important to note that PhA is a theoretical concept that has not been studied in a live cellular model. For this reason, researchers suggest that PhA is more an indicator of cellular mass rather than directly reflecting cellular health as currently interpreted [15].

Previous studies, such as those by Algül and Özçelik (2022) [34] and Kutáč (2014) [41], have shown similar effects of hydration status on body composition measurements, particularly in changes in fat mass and extracellular water. Our findings align with these studies, suggesting that acute dehydration leads to misinterpretation of body fat changes by BIA devices. In other studies aiming to determine changes in body composition through water intake, differences in muscle mass are not reported, but an increase in body fat is observed [33,41,42,43]. It appears that changes in body weight due to water loss or intake are interpreted by the BIA equipment as changes in body fat, decreasing the body fat when weight is lost through sweat and increasing body fat when water is ingested [33,44]. The inconsistency reported in the studies appears to be a limitation of BIA devices, resulting in an inaccurate estimation due to the lack of direct measurement for calculating body fat.

No differences were found in muscle mass in this study. Previous investigations reported results similar to those of this study, mentioning that subjects with greater muscle mass present a higher PhA compared to people with the same characteristics but with less muscle mass [45]. In addition, evidence suggests no significant differences between the InBody 770 (used in this study) and the gold-standard DXA in fat percentage and muscle mass in physically active men [46,47]. Thus, InBody 770 is likely a reliable equipment for measuring these variables; however, it is not recommended to determine short-term changes in some body composition variables.

Another critical aspect of this study is that no differences were found in total and intracellular body water. Nevertheless, it is assumed that the InBody 770 accurately detects these changes in body water. Similar to this study, Algül and Özçelik (2022) [34], Kutáč (2014) [41], and Ugras (2020) [42] did not report changes in TBW using BIA models: TBF 300A, MC-190, BC-418, and TBF 300A, respectively. In the first two studies, bioimpedance was analyzed after water intake, while in the last study, the subjects were passively dehydrated, not with exercise. Researchers such as Dixon et al. (2006, 2009) [35,36] mention that losing body water significantly influences body fat values but not total body water.

Changes in ECW could be due to hypertonic dehydration (i.e., the amount of water lost is greater than Na^+^ loss) due to prolonged exposure to heat [48]. Decreasing extracellular water and not Na^+^ could cause electrical bioimpedance to determine that water increases in this compartment because Na^+^ is a good conductor of electrical current, and there is a considerable amount of this mineral in the extracellular compartment [49,50]. Nevertheless, research has not reported differences in body composition before and after ingesting isotonic drinks compared to drinking water [35,36,51,52].

Despite finding differences in weight, fat mass, extracellular water, and phase angle, the Bland–Altman plots and confidence limits showed a small and constant bias between the two measurements in all the body composition variables analyzed (BW, FM, SMM, TBW, ICW, ECW, and PhA), indicating that the BIA equipment provides accurate information. These results provide good agreement between measurements, making the measurement reliable after mild passive dehydration.

This study found differences in the pre- and post-test phase angle scores (Δ = 0.09). However, considering that the CV, SWC, and MAPE provided minimal values, this difference is irrelevant from a clinical perspective. Dehydration may increase the concentration of electrolytes in the extracellular fluid, which can affect the impedance readings and consequently the calculated PhA. The loss of intracellular water might cause cells to become more concentrated, thus altering their electrical properties. The same happened for the FM and ECW variables. It is important to note that no studies were found that analyze changes in the phase angle through loss of body weight due to passive dehydration. 

### Limitations and Future Directions

A limitation of the present study is that we did not determine hydration status through plasma osmolality, which is considered the gold standard. However, the refractometry method used in this study is one of the most widely used and it is the best option when laboratory tests are inaccessible [53]. In addition, it is recommended that blood parameters be reported to provide an accurate idea of fluid exchange and mineral quantification. Future studies should consider analyzing body composition in different states of hydration in the short term, comparing variables with the dual-energy X-ray absorptiometry (DEXA) method [54]. Similarly, a thorough analysis of the relationship between phase angle and body water should be conducted, as it is one of the BIA variables that is not frequently reported. Although not determining plasma osmolality is a limitation of this study, a strength is emphasized in achieving that all participants arrived under the same hydration conditions, which is a crucial factor in BIA studies of this nature. 

For future studies with passive dehydration designs, it is recommended to determine changes in body water across different segments (e.g., legs, arms, and trunk). Quantifying and analyzing these values can help clarify TBW, FM, and ICW estimations using BIA. Designing studies with women would be interesting as most studies in this area focus on apparently healthy males aged between 20 and 30 years.

It is recommended that the relationship or potential effect between total body water and changes in phase angle under thermal stress in physically active individuals, especially in women, individuals with excess body fat (overweight or obesity), and those with chronic diseases, be investigated. These populations are susceptible to acute changes in body composition [55]. This study’s findings are based on a small sample of young, healthy, physically active men, which limits the generalizability to other populations, such as women, older adults, or those with chronic health conditions. Similarly, it is advisable to analyze these potential changes in different body segments [56].

A strength of this study is that all subjects were presented under the same hydration conditions, ensuring control and standardization of the most critical study variables. Studies analyzing changes in body composition through BIA after passive dehydration are rare. Most studies that analyze fluid changes do so through water intake [33,41,57], while other researchers focus on chronic dehydration or acute dehydration through exercise [14,58].

## 5. Conclusions

In young men, a slight loss of body water (Δ = 0.76 ± 0.34 kg) resulted in minor shifts in body composition measurements obtained through electrical bioimpedance. Specifically, passive dehydration led to a decrease in body fat mass and an increase in extracellular water and phase angle. Nonetheless, these changes had a negligible effect on the accuracy of the equipment, rendering them clinically insignificant. Therefore, it can be inferred that the body composition apparatus yields dependable information about alterations caused by passive dehydration. Nevertheless, precautions are recommended when conducting BIA assessments, especially in clinical settings. Standardizing hydration status can provide more accurate information when monitoring evaluations.

## Figures and Tables

**Table 1:** Descriptive and inferential statistics summary table for individuals undergoing five hours of passive dehydration under thermal stress (*n* = 25).

Variable	Pre-Test	Post-Test	MDiff	*p*	Effect Size	95% CI for Effect Size		*n*	%	95% CI MDiff	
						Lower	Upper			Lower	Upper
Body mass (kg)	76.7 ± 15.9	75.9 ± 15.8 a	−0.8	<0.001	2.25	1.50	2.99	24	96	−1.4	−0.1
Fat mass (kg)	18.4 ± 8.0	17.5 ± 8.1 a	−0.9	<0.001	0.83	0.62	0.93	25	100	0.2	−0.4
SMM (kg)	33.1 ± 5.7	33.0 ± 5.9 a	0.1	0.463	−0.15	−0.54	0.25	24	96	−1.0	1.1
TBW (L)	42.5 ± 6.8	42.7 ± 6.6 b	0.2	0.059	−0.43	−0.72	−0.02	25	100	−1.1	1.5
ICW (L)	26.9 ± 4.4	27.0 ± 4.2 a	0.1	0.492	−0.14	−0.53	0.26	24	96	−0.7	0.8
ECW (L)	15.6 ± 2.5	15.7 ± 2.4 b	0.1	0.021	−0.58	−0.82	−0.17	25	100	−0.5	0.7
PhA (°)	6.76 ± 0.6	6.85 ± 0.6 a	0.1	0.005	−0.61	−1.04	−0.18	25	100	−0.2	0.4

Note. Data are expressed as mean (M) and standard deviation (SD); *n*: number of values within the 95% CI of mean differences between pre-test and post-test: percentage of the values within the LoA; MDiff: mean differences between pre-test and post-test; SMM: skeletal muscle mass; TBW: total body water; ICW: intracellular water; ECW: extracellular water; PhA: phase angle. a: mean comparison by Student’s *t*-test; b: mean comparison by Wilcoxon test. For the Student’s *t*-test, effect size is given by Cohen’s d. For the Wilcoxon test, effect size is given by the matched rank biserial correlation.

**Table 2:** Reliability of body composition scores as shown by the typical error of the measurement (TEM), the coefficient of variability (CV), the smallest worthwhile change (SWC), and the mean absolute percentage error (MAPE) for individuals completing the protocol (*n* = 25).

Variable	TEM	CV (%)	SWC	MAPE (%)
BM (kg)	0.24	0.31	0.07	1.0
FM (kg)	0.62	3.43	0.17	1.6
SMM (kg)	0.38	1.14	0.11	1.6
TBW (L)	0.48	1.12	0.13	1.3
ICW (L)	0.28	1.05	0.08	1.2
ECW (L)	0.21	1.36	0.06	1.6
PhA (°)	0.10	1.52	0.03	2.1

BM: body mass; FM: fat mass; SMM: skeletal muscle mass; TBW: total body water; ICW: intracellular water; ECW: extracellular water; PhA: phase angle.

## Data Availability

The data presented in this study are available on request from the corresponding author. The data are not publicly available due to the project in progress and privacy.
